# Retrospective evaluation of active tuberculosis cases in terms of chemoprophylaxis indication: A cross-sectional study

**DOI:** 10.1097/MD.0000000000043202

**Published:** 2025-07-04

**Authors:** Ezgi Ongoz, Olcay Aycicek, Yilmaz Bülbül, Funda Öztuna, Tevfik Özlü

**Affiliations:** a Department of Chest Diseases, Karadeniz Technical University Faculty of Medicine, Trabzon, Turkey.

**Keywords:** chemoprophylaxis, latent tuberculosis infection, tuberculosis

## Abstract

In this study, we retrospectively evaluated the indications for chemoprophylaxis in active tuberculosis cases and reviewed the risk of latent tuberculosis infection (LTBI) progressing to active disease. Along with our decisions regarding LTBI treatment indications, we aimed to determine whether there were any shortcomings in this area. This study included 422 tuberculosis patients who were registered at the Provincial Central Tuberculosis Control Dispensary and diagnosed between 2016 and 2020. This was a single-center retrospective clinical study. These patients were evaluated in terms of latent tuberculosis treatment indication criteria in the Tuberculosis Diagnosis and Treatment Guide before they became patients with tuberculosis. All radiological images of the patients were evaluated. A total of 422 patients were included in this study. Of these, 147 (34.8%) were women, and 275 (65.2%) were men. When examining the distribution of patients according to the indications for latent tuberculosis infection treatment specified in the Ministry of Health’s 2019 Tuberculosis Diagnosis and Treatment Guide, it was found that 6 (1.4%) patients were in contact with infectious cases, with 3 (50.0%) of these patients under 34 years old and 3 (50.0%) 35 years old or older. Prophylactic treatment was administered to 1 patient (33.3%) in the < 34 years age group. One patient with conversion was identified in the last 2 years, and it was noted that this patient received prophylaxis. Of the 153 patients with radiological findings compatible with tuberculosis sequelae, three (2.0%) received prophylaxis. According to the tuberculosis diagnosis and treatment guide, after adjusting for patients with common criteria, 164 (38.8%) patients were indicated for LTBI treatment. However, prophylaxis was only provided to 7 (4.2%) of these patients. The low rate of chemoprophylaxis observed in our study highlights the importance of diagnosing and monitoring LTBI. The data obtained will contribute to the diagnosis and treatment of LTBI.

## 1. Introduction

Tuberculosis (TB) is an important disease caused by infection with *Mycobacterium tuberculosis* (MTB) bacillus, commonly acquired through the respiratory route, and has affected societies socially and economically for many years.^[1]^ It continues to be a significant infectious disease that causes the most deaths worldwide. Approximately one-quarter of the global population is estimated to be infected with MTB.^[2]^ A condition characterized by a sustained immune response against MTB antigens without any clinical symptoms is referred to as latent tuberculosis infection (LTBI).^[3]^ It is believed that about one-quarter of the world’s population is believed to have LTBI. The vast majority of infected individuals are not sick or infectious; however, it is thought that individuals infected with MTB have a lifetime risk of developing tuberculosis disease ranging from 5% to 15%.^[4]^ This activation often occurs within the first 5 years following the initial infection. Conditions that can lead to the development of active disease from LTBI include HIV (human immunodeficiency virus) infection, malnutrition, alcohol and tobacco use, diabetes, autoimmune diseases, use of immunosuppressive agents, and organ transplantation.^[4,5]^

In terms of controlling tuberculosis infection, it is crucial not only to identify and treat active cases, but also to detect LTBI and provide preventive treatment to individuals at high risk of developing active TB. Accurately determining who should receive preventive treatment is important. As LTBI often presents subclinically, it is not possible to directly identify it; therefore, tests that measure the adaptive immune response generated in the body against LTBI are utilized. Although new biomarkers and measurement studies are ongoing, the most commonly used tests for diagnosing LTBI are the tuberculin skin test (TST) and interferon-gamma release assay (IGRA).^[6]^

The treatment of LTBI, which is not an active and symptomatic disease, is indicated because of the risk of progression to active TB. With LTBI treatment, progression to active TB can be prevented by 60% to 90%. Instead of treating a TB patient after the development of an active disease, it is a more rational and acceptable approach to treat LTBI cases that have a high risk of reactivation before the disease develops. This approach is also more cost-effective because of the use of fewer medications, resulting in fewer treatment side effects, minimal resistance issues, and prevention of the spread of TB infection within the community, thus aiding in the control of tuberculosis.^[7]^ In practice, it is assumed that the protection lasts for a lifetime; however, LTBI treatment does not completely prevent reinfection. In Turkey, the groups recommended for LTBI treatment are outlined in the Ministry of Health’s 2019 Tuberculosis Diagnosis and Treatment Guide.^[8]^

This study aimed to retrospectively evaluate the indications for chemoprophylaxis in active tuberculosis cases, review the risk of latent tuberculosis infection progressing to active disease, and assess our decisions regarding LTBI treatment indications to identify any shortcomings in this area and to contribute to tuberculosis control.

## 2. Methods

The study commenced after obtaining approval from the Karadeniz Technical University Ethics Committee (File No: 2021-231). This was a single-center, retrospective clinical study. A total of 422 tuberculosis patients aged 18 years and above who were registered at the Provincial Tuberculosis Control Dispensary and diagnosed between 2016 and 2020 were included in the study.

Exclusion criteria were defined as patients under 18 years of age and those whose examination and laboratory information could not be accessed through the hospital system or e-Nabiz (e-Nabiz; a personal health record system implemented by the Ministry of Health of the Republic of Turkey, allowing citizens to view and manage their personal health information via an Internet-based service and mobile application).

Data collected included patient age, sex, occupational risks, comorbidity status, tobacco and substance use in the 2 years prior to diagnosis, use of immunosuppressive medications and their types, radiological findings, previous evaluation for chemoprophylaxis, results of PPD (purified protein derivative) and Quantiferon tests, presence of BCG vaccine (Bacillus Calmette-Guérin) scar, contact status with infectious tuberculosis cases, age at contact, type of contact, duration of contact, presence of conversion, and whether they received preventive treatment. All radiological images of the patients were evaluated. Since this was a retrospective study, informed consent was not obtained from the patients.

Statistical analysis was performed using the Statistical Package for the Social Sciences software version 23.0. Descriptive statistics of the evaluation results were presented as counts and percentages for categorical variables and as mean, standard deviation, minimum, and maximum for numerical variables. The Kolmogorov–Smirnov and Shapiro–Wilk tests were used to test the normal distribution of numerical variables. When the numerical data did not show a normal distribution, the Mann–Whitney *U* test was used for the analysis. Chi-square and Fisher’s exact tests were used to compare categorical data. Correlation coefficients and statistical significance between non-normally distributed numerical variables were examined using Spearman test. Statistical significance was set at *P* < .05.

## 3. Results

In total, 422 patients were included in this study. Of these, 147 (34.8%) were women, and 275 (65.2%) were men. The average age of the included patients was 53.27 ± 18.71 years, with an average age of 49.59 ± 21.01 years for female patients and 55.23 ± 17.08 years for male patients (*P* = .006). Prophylaxis was applied to only 7 (1.6%) patients. There were 12 foreign patients (2.8%), none of whom received prophylaxis. The demographic data of the patients who received prophylaxis and those who did not are presented in Table 1.

**Table 1:** Demographic data of patients.

	Patients who did not receive chemoprophylaxis (n = 415)	Patients who received chemoprophylaxis (n = 7)	Total (n = 422)
Gender			
Female	144 (98.0%)	3 (2.0%)	147 (34.8%)
Male	271 (98.5%)	4 (1.5%)	275 (65.2%)
Age	53.39 ± 18.68	46.14 ± 20.90	53.27 ± 18.71
Occupational risks			
Healthcare worker	7 (1.7%)	0 (0.0%)	7 (1.7%)
Nursing home, prison staff	7 (1.7%)	0 (0.0%)	7 (1.7%)
Marble, stone work	1 (0.2%)	0 (0.0%)	1 (0.2%)
Inorganic dust exposure	12 (2.9%)	0 (0.0%)	12 (2.8%)
Mining work	4 (1.0%)	0 (0.0%)	4 (0.9%)
Heating technician	0 (0.0%)	0 (0.0%)	0 (0.0%)
Comorbidity			
None	206 (49.6%)	1 (14.3%)	207 (49.1%)
Vasculitis	2 (0.5%)	0 (0.0%)	2 (0.5%)
HIV	2 (0.5%)	0 (0.0%)	2 (0.5%)
Chronic kidney disease	19 (4.6%)	1 (14.3%)	20 (4.7%)
Ocular disease	1 (0.2%)	0 (0.0%)	1 (0.2%)
Inflammatory bowel disease	1 (0.2%)	0 (0.0%)	1 (0.2%)
DM	56 (13.5%)	1 (14.3%)	57 (13.5%)
Transplantation	0 (0.0%)	0 (0.0%)	0 (0.0%)
Rheumatic disease	11 (2.7%)	2 (28.6%)	13 (3.1%)
Interstitial lung disease	1 (0.2%)	0 (0.0%)	1 (0.2%)
Malignancy	30 (7.2%)	2 (28.6%)	32 (7.6%)
Multiple sclerosis	1 (0.2%)	0 (0.0%)	1 (0.2%)
Skin disease	0 (0.0%)	1 (14.3%)	1 (0.2%)
Silicosis	0 (0.0%)	0 (0.0%)	0 (0.0%)
Gastrectomy/Jejuno-ileal bypass	3 (0.7%)	0 (0.0%)	3 (0.7%)
Substance use (use in the last 2 years prior to tuberculosis diagnosis)			
Tobacco	260 (62.7%)	2 (28.6%)	262 (62.1%)
Alcohol	10 (2.4%)	1 (14.3%)	11 (2.6%)
Drugs	2 (0.5%)	0 (0.0%)	2 (0.5%)
Foreign nationals	12 (2.9%)	0 (0.0%)	12 (2.8%)

DM = diabetes mellitus, HIV = human immunodeficiency virus.

Four (57.1%) patients who received prophylaxis were tested with the PPD test, and the mean value was 17.25 ± 4.34 mm. In the group that did not receive prophylaxis, 43 (10.4%) patients were tested, and the average PPD value was 14.79 ± 10.80. The Quantiferon test was applied to only 3 patients in the group that did not receive prophylaxis, and the test results were negative for 2 patients, while 1 patient tested positive (Table 2).

**Table 2:** Patients’ PPD and quantiferon test data.

	Patients not receiving chemoprophylaxis (n = 415)	Patients receiving chemoprophylaxis (n = 7)	Total (n = 422)
PPD evaluation	43 (10.4%)	4 (57.1%)	47 (11.1%)
PPD value	14.79 ± 10.80	17.25 ± 4.34	15.00 ± 10.40
Presence of BCG scar	254 (61.2%)	7 (100.0%)	261 (61.8%)
Quantiferon test			
Positive	1 (0.2%)	0 (0.0%)	1 (0.2%)
Negative	2 (0.5%)	0 (0.0%)	2 (0.5%)
Not done	412 (99.3%)	7 (100.0%)	419 (99.3%)

BCG = Bacillus Calmette-Guérin, PPD = purified protein derivative.

Six patients with a history of contact with a person with infectious pulmonary tuberculosis in the last 2 years before the diagnosis of tuberculosis were identified. Of these 6, 1 (14.3%) received prophylaxis treatment. When the distribution of the contacts according to age groups was analyzed, there were 3 (50%) people in the age range of 16 to 35 years and 2 (40.0%) people did not receive chemoprophylaxis, while 1 (60.0%) person received chemoprophylaxis. Three individuals aged 36 years and over were identified and none of these individuals received prophylaxis. All contacts (100.0%) were considered as close household contacts. In addition, all patients (100.0%) had contact in a closed environment for more than 8 hours. In terms of the criterion of repeated weekly contact for more than 4 hours, 80.0% (4 individuals) of patients who did not receive chemoprophylaxis and 100.0% (1 individual) of patients who received chemoprophylaxis were in this group. In general, the proportion of individuals with this contact duration was 83.3%. Conversion rate, which is sone of the markers of infection, was determined as 1 (0.2%) in the group not receiving chemoprophylaxis, while no conversion was detected in the group receiving chemoprophylaxis (Table 3).

**Table 3:** History of contact with infectious pulmonary tuberculosis case in the last 2 years prior to tuberculosis diagnosis.

	Patients not receiving chemoprophylaxis, n = 5 (1.2%)	Patients receiving chemoprophylaxis, n = 1 (14.3%)	Total, n = 6 (1.4%)
Age groups of contacts			
16–35 age	2 (40.0%)	1 (100.0%)	3 (50%)
36 and older	3 (60.0%)	0 (0.0%)	3 (50%)
Household close contact	5 (100.0%)	1 (100.0%)	6 (100.0%)
Contact for more than 8 h in an enclosed space	5 (100.0%)	1 (100.0%)	6 (100.0%)
Repeated contact for more than 4 h per week	4 (80.0%)	1 (100.0%)	5 (83.3%)
Conversion	1 (0.2%)	0 (0.0%)	1 (0.2%)

Conversion: TST becomes positive with an increase of at least 6 mm from negative to positive or an increase of 10 mm or more even in the absence of positivity.

When evaluated according to immunosuppressive drug use, 52 (12.3%) patients used one of these medications. Among patients who did not receive prophylaxis, 2 (0.5%) used anti-tumor necrosis factor (anti-TNF), 1 (0.2%) used monoclonal antibody drugs, 12 (2.9%) used chemotherapeutics, 16 (3.9%) used systemic steroids, 3 (0.7%) used antimetabolite drugs, and 12 (2.9%) used other types of immunosuppressive drugs not listed in these categories. The distribution of patients who did and did not receive prophylaxis based on their immunosuppressive status is shown in Table 4.

**Table 4:** Distribution of patients according to immunosuppressive drug use status.

Immunosuppressive drug use	Patients not receiving chemoprophylaxis (n = 415)	Patients receiving chemoprophylaxis (n = 7)	Total (n = 422)
Anti-TNF	2 (0.5%)	2 (28.6%)	4 (0.9%)
Monoclonal antibody	1 (0.2%)	0 (0.0%)	1 (0.2%)
Chemotherapeutic	12 (2.9%)	1 (14.3%)	13 (3.1%)
Systemic steroid	16 (3.9%)	1 (14.3%)	17 (4.0%)
Antimetabolites (methotrexate, fluorouracil)	3 (0.7%)	1 (14.3%)	4 (0.9%)
Other	12 (2.9%)	1 (14.3%)	13 (3.1%)

Anti-TNF = anti-tumor necrosis factor.

No radiological abnormalities were detected in 51 patients (12.1%). These patients had non-pulmonary tuberculosis cases. The number of patients with findings compatible with sequelae of tuberculosis on chest radiography was 153 (36.3%). The most common finding was a calcified nodule, observed in 87 (20.6%) patients, while the least observed finding was an apical cap, seen in 2 (0.5%) patients. Among the patients who received prophylaxis, 1 (14.3%) had a normal chest radiograph, 3 (42.9%) had a calcified nodule, and 1 (14.3%) had a fibrotic sequela. The remaining patients had findings consistent with active tuberculosis. In the group that did not receive prophylaxis, 34 (8.2%) patients had granulomas, 36 (8.7%) had calcified hilar LAP, 84 (20.2%) had calcified nodules, 44 (10.6%) had fibronodular sequelae, and 2 (0.5%) had an apical cap (Table 5).

**Table 5:** Distribution of patients according to radiological findings.

Radiological findings	Patients not receiving chemoprophylaxis (n = 415)	Patients receiving chemoprophylaxis (n = 7)	Total (n = 422)
Normal	50 (12.0%)	1 (14.3%)	51 (12.1%)
Findings compatible with sequel of tuberculosis			
Granuloma	34 (8.2%)	0 (0.0%)	34 (8.1%)
Calcified hilar lymphadenopathy	36 (8.7%)	0 (0.0%)	36 (8.5%)
Calcified pulmonary nodule	84 (20.2%)	3 (42.9%)	87 (20.6%)
Fibronodular sequel	44 (10.6%)	1 (14.3%)	45 (10.7%)
Apical cap	2 (0.5%)	0 (0.0%)	2 (0.5%)
Findings compatible with active tuberculosis			
Mediastinal lymphadenopathy	50 (12.0%)	1 (14.3%)	51 (12.1%)
Miliary pattern	2 (0.5%)	0 (0.0%)	2 (0.5%)
Tree-in-bud	32 (7.7%)	0 (0.0%)	32 (7.6%)
Cavitary lesion	100 (24.1%)	2 (28.6%)	102 (24.2%)
Consolidation	114 (27.5%)	3 (42.9%)	117 (27.7%)
Pulmonary nodule	65 (15.7%)	1 (14.3%)	66 (15.7%)
Pleural effusion	59 (14.2%)	0 (0.0%)	59 (14.0%)

The distribution of patients was analyzed according to the latent tuberculosis infection treatment indications specified in the Turkish Ministry of Health’s 2019 Tuberculosis Diagnosis and Treatment Guidelines (Table 6). It was found that 6 (1.4%) patients had been in contact with infectious tuberculosis cases; of these, 3 (50.0%) were under 34 years old, and 3 (50.0%) were 35 years or older. All patients under 34 years of age were evaluated for prophylaxis, and 1 (33.3%) was recommended prophylaxis and received treatment. PPD test information for this patient was not available. Among the immunosuppressed LTBI-positive patients over 35 years old, 1 (33.3%) patient had malignancy, prophylaxis was not recommended, and the patient did not receive treatment.

**Table 6:** Distribution of patients according to latent tuberculosis infection (LTBI) treatment indications according to the ministry of health’s 2019 tuberculosis diagnosis and treatment guidelines.

	Number of patients (n/%)	Those evaluated for prophylaxis (n/%)	Those recommended for prophylaxis (n/%)	PPD value (mm)	Those who received prophylaxis (n/%)
(1) Contacts with active TB patients	6 (1.4%)	6 (100%)	1 (16.7%)	15.00 ± 10.409	1 (16.7%)
Babies born to mothers with TB	–	–		–	–
People under 34	3 (50.0%)	3 (100%)	1 (33.3%)	–	1 (33.3%)
Over 35, LTBI (+), immunocompromised	1 (16.6%)	1 (100%)	0 (0.0%)	30 ± 0.0	0 (0.0%)
(2) PPD test (+), persons under 15 years of age	–	–	–	–	–
(3) Those who have had a TST conversion in the last 2 years	1 (0.2%)	1 (100%)	1 (100%)	20 ± 0.0	1 (100%)
(4) Those with findings consistent with TB sequelae on chest X-ray	153 (36.3%)	3 (2.0%)	3 (2.0%)	20.71 ± 8.389	3 (2.0%)
(5) Immunocompromised, LTBI (+) individuals					
HIV-positive people	0 (0.0%)	0 (0.0%)	0 (0.0%)	0 (0.0%)	0 (0.0%)
People who will start anti-TNF treatment	4 (0.9%)	2 (50%)	2 (50%)	20 ± 3.559	2 (50%)
Those receiving corticosteroid treatment	5 (1.1%)	1 (20.0%)	1 (20.0%)	13.71 ± 10.144	1 (20.0%)
Dialysis patients	2 (0.4%)	1 (50.0%)	1 (50.0%)	21.50 ± 4.95	1 (50.0%)
Organ or hematological transplant recipient/donor candidates	0 (0.0%)	–		–	–
Silicosis patients	0 (0.0%)	–		–	–
Total number†	169	12	10	–	9

Anti-TNF = anti-tumor necrosis factor, HIV = human immunodeficiency virus, LTBI = latent tuberculosis infection, PPD = purified protein derivative, TB = tuberculosis, TST = tuberculin skin test.

† The number of patients who are indicated for LTBI treatment, after corrections were made for patients with common criteria, was calculated as 164 (38.8%), and it was determined that prophylaxis was given to 7 (1.6%) of these patients.

A patient with conversion in the last 2 years was identified, and prophylaxis was recommended. Among the 153 patients with findings compatible with tuberculosis sequelae on chest X-rays, 136 (88.9%) were over 35 years old and 17 (11.1%) were under 35. Among these patients, 3 (2.0%) were evaluated for prophylaxis and treatment was recommended, and all received prophylaxis. Of the patients who received prophylaxis, 2 were under 35 years of age and 1 was over 35 years of age.

Among the immunosuppressed patients, 4 (0.9%) were on anti-TNF therapy, 5 (1.1%) were using systemic steroids, and 2 (0.4%) were on dialysis. Two (50%) patients in the anti-TNF group, 1 (20.0%) patient in the systemic steroid group, and 1 (50.0%) patient in the dialysis group received prophylaxis. There were no LTBI-positive HIV patients, transplant patients, or silicosis patients.

According to the corrected criteria for patients eligible for LTBI treatment, 164 patients (38.8%) met the inclusion criteria. However, only 7 (4.2%) of these patients received prophylaxis. All patients receiving prophylaxis received isoniazid treatment, and treatment noncompliance was present in only 1 patient (Table 6).

According to the 2015 Latent Tuberculosis Guide of the World Health Organization (WHO), LTBI screening is not considered an absolute indication but should be considered for certain patient groups. When evaluating the patients in our study based on these groups, 7 (1.7%) were nursing homes or prison workers, 7 (1.7%) were healthcare workers, 12 (2.9%) were immigrants, and 2 (0.5%) were substance abusers. Information on whether any of the patients were homeless was not obtained. None of these patient groups were evaluated for prophylaxis, nor was any treatment administered (Table 7).

**Table 7:** Distribution of patients according to the WHO guidelines on the management of latent tuberculosis infection, 2015, for those recommended for prophylaxis evaluation.

	Number of patients (n/%)	Those evaluated for prophylaxis (n/%)	Those recommended for prophylaxis (n/%)	PPD value (mm)	Those who received prophylaxis (n/%)
Nursing home, prison staff	7 (1.7%)	0 (0.0%)	0 (0.0%)	–	0 (0.0%)
Healthcare workers	7 (1.7%)	0 (0.0%)	0 (0.0%)	–	0 (0.0%)
Immigrants from high TB burden countries	12 (2.9%)	0 (0.0%)	0 (0.0%)	–	0 (0.0%)
Homeless individuals	0 (0.0%)	–	–	–	–
Substance abusers (drug users)	2 (0.5%)	0 (0.0%)	0 (0.0%)	25 ± 0.0	0 (0.0%)

WHO = World Health Organization.

According to the WHO 2015 LTBI guidelines, evaluation of groups not recommended for LTBI screening revealed that 57 (13.5%) patients had diabetes mellitus. Among these, 2 (3.5%) were evaluated for prophylaxis, 1 (1.8%) was recommended prophylaxis, and prophylaxis was administered the diabetic patients, while 5 (8.8%) were using immunosuppressive medications. These included 1 (1.8%) patient on anti-TNF therapy, 1 (1.8%) patient on chemotherapy, 2 (3.5%) patients on systemic steroids, and 1 (1.8%) patient on other immunosuppressive treatments. It was found that the patient who received prophylaxis was undergoing chemotherapy, whereas the patient on anti-TNF therapy did not receive prophylaxis, and 262 (62.1%) patients used tobacco. Among them, 5 (1.9%) were evaluated for prophylaxis and 2 (0.7%) were recommended and received prophylaxis. Of the patients who received prophylaxis, one was undergoing chemotherapy, and the other was a dialysis patient; 11 (2.6%) patients consumed alcohol. Among these, 2 (18.0%) were evaluated for prophylaxis. One of these patients received anti-TNF therapy, and the other received methotrexate.

## 4. Discussion

According to 2020 data from the Ministry of Health’s Tuberculosis Department, 95.6% of tuberculosis cases in our country are found in the age group of 15 years and older, and 57.2% of the cases were male, while 42.8% were female.^[9]^ In our study, the distribution of patients was similar to that reported in the literature, with 147 (34.8%) females and 275 (65.2%) males. Research on the gender association of TB and LTBI shows that men have a higher prevalence than women.^[10]^ Among the 7 patients who received chemoprophylaxis in our study, 3 (2.0%) were female and 4 (1.5%) were male.

Close contact with an active tuberculosis patient and the duration of this contact are significant risk factors for LTBI transmission. Healthcare workers are also at increased risk, which correlates with the intensity and duration of exposure. In a study conducted in Uganda, the prevalence of tuberculosis among 396 healthcare workers was 57%.^[11]^ In a study in the United States, 5% of healthcare workers tested positive for IGRA and 3% for TST during initial screening. After repeated evaluations, a transition from negative to positive results was observed in 4% of IGRA tests and 0.7% of TST tests.^[12]^ In our study, the number of healthcare workers was 7 (1.7%). None of these patients had previously been evaluated for prophylaxis, nor had they received treatment or underwent PPD/quantiferon testing. Regular PPD screening and conversion follow-up in this risk group will contribute to early diagnosis and treatment. The risk of developing tuberculosis in healthcare workers is 0.6 to 2 times higher than in the general population. Nurses, chest and infectious disease specialists, doctors, pathologists and laboratory workers constitute the most risky group. It is difficult to completely eliminate this risk, but respiratory and environmental precautions should be taken to minimize tuberculosis transmission, for example, staining for microscopic examination or culture cultivation should be performed in special areas of the hospital and using protective equipment, or ventilation and filtering methods should be used to prevent the concentration and spread of infectious particles in the treated area. Administratively, each health institution should educate its staff about TB, its transmission routes and prevention methods, and periodically assess and monitor them for latent TB. All these measures against TB transmission should be transformed into written protocols and regularly audited by higher centers. Unfortunately, inspection and sanction mechanisms are often lacking and this can lead to employees not being regularly examined. The lack of adequate training of healthcare workers on the subject also causes them to be negligent in this regard.

The risk of tuberculosis and latent TB is a significant concern for personnel working in nursing homes or prisons. In our study, 7 patients (1.7%) who were nursing homes or prison workers did not receive preventive treatment. This finding suggests that tuberculosis screening programs are insufficient. Studies indicate that the risk of tuberculosis is high in prisons and that regular screening can prevent transmission. Similarly, it is emphasized that prophylaxis treatment for prison staff should be increased.^[13,14]^ The risk of tuberculosis transmission is especially high in places such as these, and annual screening of personnel working in these places for latent tuberculosis infection or active tuberculosis development and regular training to prevent the risk of transmission will contribute to tuberculosis control.

Tobacco, alcohol, and substance use increases the frequency of tuberculosis and the risk of latent TB progressing to active tuberculosis.^[15,16]^ Yu et al in their study found that smoking was associated with a higher prevalence of pulmonary tuberculosis compared to those who had quit smoking.^[17]^ In our study, 262 (62.1%) patients were smokers, and 5 (1.9%) were evaluated for prophylaxis, with 2 (0.7%) receiving prophylaxis. Of the patients who received prophylaxis, one was undergoing chemotherapy and the other was undergoing dialysis. The WHO 2015 Latent Tuberculosis Guidelines include individuals who use tobacco and alcohol among a group of people not recommended for LTBI screening.^[18]^ In our study, the prevalence of tobacco use was higher in patients who developed tuberculosis. Numerous epidemiologic studies and meta-analyses have shown that active and passive smoking are independent risk factors for tuberculosis infection, tuberculosis reactivation, primary tuberculosis progression, development of cavitary disease and disease exacerbation and mortality.^[19]^ We believe that these individuals should be included in the group that has undergone LTBI screening.

According to 2020 data from the Tuberculosis Department, 15.9% of tuberculosis cases in our country are individuals born outside Turkey. The number of foreign nationals in our study was lower than that of our country. Studies have shown that treatment nonadherence is common among migrants.^[20]^ This poses a risk in terms of the development of resistance and transmissibility. Due to the disease burden in foreign nationals and migrant populations, latent TB infections are reactivated and become a public health issue.^[21]^ The WHO 2015 Latent Tuberculosis Guidelines include migrants from countries with a high TB burden as a group for which LTBI screening should be considered. However, none of the foreign nationals in our study was evaluated for prophylaxis. It is important to include migrants in screening programs for latent tuberculosis and to educate them about tuberculosis and its transmission routes, especially in countries receiving migrants from regions with a high incidence of tuberculosis. Migrants’ access to health services can sometimes be difficult due to language barriers or legal situations. Therefore, the establishment of a system to facilitate access to health services will lead to a higher number of people participating in the screening system.

Immunocompromised patients are at risk of latent tuberculosis infection developing into active disease. In our study, 164 patients were immunocompromised (38.8%). However, only 7 (4.2%) of these patients received prophylaxis. Despite the indication for prophylactic treatment, the very low number of patients who received treatment indicates that the management of latent tuberculosis infection is inadequate.

In our study, 52 patients (12.3%) used immunosuppressive drugs. Among patients who did not receive prophylaxis, 2 (0.5%) used anti-TNF drugs, 1 (0.2%) used monoclonal antibodies, 12 (2.9%) used chemotherapeutic drugs, 16 (3.9%) used systemic steroids, 3 (0.7%) used antimetabolite drugs, and 12 (2.9%) used other types of immunosuppressive drugs. A review of the literature shows that most studies examining the relationship between latent tuberculosis and immunosuppressive drugs have focused on the anti-TNF drug group.The risk of tuberculosis is high among patients receiving anti-TNF-α treatment and studies on this subject reveal that these patients should be monitored more carefully.^[22]^ However, in our study, the most commonly used immunosuppressive drugs were systemic steroids, followed by chemotherapeutic drugs.

It has been repeatedly proven that the risk of tuberculosis infection increases in patients using anti-TNF-α drugs.^[23]^ A literature review revealed a study by Hanta et al in 2008, in which 179 patients receiving TNF-α antagonists were followed for 3 years, and TB developed in 3 patients, resulting in an incidence rate of 1.5%.^[24]^ In our study, the number of patients with LTBI using anti-TNF in the last 2 years was 4 (0.9%). Of these patients, 2 (28.6%) had received prophylaxis previously, while 2 (0.5%) did not. When the relationship between latent tuberculosis and anti-TNF use was evaluated, a study by Jauregui-Amezega et al involving 423 inflammatory bowel disease patients found an LTBI positivity rate of 6.9%.^[25]^ In our study, however, the number of LTBI (+) patients receiving anti-TNF treatment was 4 (0.9%), which is a lower percentage. Although the number of patients receiving anti-TNF treatment in our study was small, we observed that, in daily practice, the number of such patients is considerably higher, and this frequently used drug group requires close monitoring because of its increased risk of developing active tuberculosis.

Corticosteroid treatment is frequently used in our country for various indications, and because of its immunosuppressive effect, which influences cytokine synthesis and lymphocyte function, it triggers many infections, particularly tuberculosis. Although there is uncertainty regarding whether low-dose steroid use increases the risk of TB, it has been established that high-dose steroid use increases the incidence of TB.^[26]^ The American Thoracic Society Consensus Report defines prolonged high-dose corticosteroid use as steroid doses equivalent to 15 mg or more of prednisone per day for 2 to 4 weeks, and these doses are considered sufficiently high.^[27]^ According to the 2019 Tuberculosis Diagnosis and Treatment Guidelines of the Ministry of Health, LTBI screening is recommended before high-dose corticosteroid use, as defined in the American Thoracic Society Consensus Report. In line with the literature, steroid use of patients participating in our study was assessed according to this definition. Although the general consensus regarding low-dose corticosteroid use is that the TB incidence is similar to that in the general population, Youssef et al reported that steroid use, regardless of dose, is associated with TB in patients with rheumatic diseases treated with corticosteroids.^[28]^ In a cohort study conducted in Canada on adults, 112,300 patients with rheumatoid arthritis were evaluated and 386 TB cases were detected. Patients receiving corticosteroids in combination with disease-modifying antirheumatic drugs or biological agents were assessed for TB. The study showed that all immunosuppressive treatment regimens, including biological agents, are associated with TB.^[29]^

Tuberculosis can occur due to immunosuppression in malignancies. A case series of cancer patients from South Korea showed that those with solid organ malignancies had a 4.7-fold higher risk of developing active TB.^[30,31]^ A patient series from Japan, which included 445 cases, demonstrated that various solid organ malignancies put patients at risk for active TB and that chemotherapy increased the risk of latent tuberculosis progressing to active disease.^[32]^ In our study, there were 13 (3.1%) patients in the chemotherapy group, and only 1 (14.3%) of these patients received prophylactic treatment. We believe that the oversight of other patients could be due to their oncology follow-up, with no referral to a pulmonology clinic.

Diseases that impair the immune system, particularly HIV, increase the development of TB in all age groups. HIV enhances the progression and spread of TB infection.^[33,34]^ In our study, there were 2 (0.5%) HIV-positive patients, and no LTBE positivity was detected in these patients. Chemoprophylaxis administered to patients with LTBE positivity plays a critical role in reducing the TB burden and has been shown to reduce the TB incidence by 30% to 50%.^[35,36]^

The risk of developing tuberculosis is 16 times higher in dialysis patients and 20 to 50 times higher in transplant recipients than that in the general population.^[37,38]^ In our study, there were 20 dialysis patients, but among the immunocompromised LTBE (+) patients, only 2 (0.4%) were dialysis patients, and 1 (50.0%) of these patients received chemoprophylaxis. It was considered that the other patient might not have received treatment because to was over the age of 35.

Many studies have reported that the risk of tuberculosis infection in diabetic patients is 2 to 4 times higher than that in the general population.^[39]^ Although the 2015 WHO Latent Tuberculosis Guide does not recommend LTBE screening for patients with diabetes mellitus, studies have shown that the risk of LTBE progressing to active tuberculosis is high in these patients. In our study, although there were 57 (13.5%) diabetic patients, only 1 (1.8%) patient was recommended and received prophylaxis. Among the diabetic patients in our study, 5 (8.8%) were using immunosuppressive drugs. The patient who received prophylaxis was found to be receiving chemotherapy, while no prophylaxis was administered to the patient using anti-TNF. Owing to the retrospective nature of our study, some patient information could not be accessed.

In our study, 153 patients with radiological findings compatible with the sequelae of tuberculosis. Among the patients who received prophylaxis, 1 (14.3%) had a normal chest radiograph, 3 (42.9%) had calcified nodules, 1 (14.3%) had a fibrotic sequela, and the remaining patients had findings consistent with active tuberculosis. In the group that did not receive prophylaxis, 34 (8.2%) patients had granulomas, 36 (8.7%) had calcified hilar lymphadenopathy, 84 (20.2%) had calcified nodules, 44 (10.6%) had fibronodular sequelae, and 2 (0.5%) had apical caps. No radiological abnormalities were found on imaging of 51 (12.1%) patients, and these patients had extrapulmonary tuberculosis. According to the 2019 Tuberculosis Diagnosis and Treatment Guide of the Ministry of Health, latent tuberculosis infection treatment is recommended for patients with chest X-ray findings compatible with TB sequelae. Despite the fact that 153 (36.3%) patients in our study had radiological findings compatible with TB sequelae, only 3 (2.0%) were administered prophylactic treatment. It is evident that the evaluation of patients with sequelae was unfortunately inadequate, and our study shows that more attention should be paid to this issue.

It is well known that contact with an infectious tuberculosis patient within the last 2 years significantly increases the risk of developing latent tuberculosis infection into an active disease. Additionally, the degree and duration of contact are important factors. In our study, 6 (1.4%) patients had a history of contact with an infectious pulmonary tuberculosis case within the last 2 years prior to the tuberculosis diagnosis. The nature of contact for all patients was close household contact for more than 8 hours. All patients in the group aged < 34 years were evaluated for prophylaxis, and 1 (33.3%) received prophylactic treatment. Information could not be obtained as to why prophylaxis was not recommended for the other 2 contacts under 34 years of age. One (33.3%) immunocompromised patient over 35 years of age was LTBI positive. This patient had malignancy, and prophylaxis was not recommended. Given the patient’s age and malignancy, a risk-benefit analysis may have been conducted, leading to the decision not to administer treatment.

If the TST is negative in contact cases, a second test should be performed 8 to 12 weeks later to rule out the possibility of a newly acquired infection. Unfortunately, delays in diagnosis and treatment can occur because of insufficient follow-up and patients not returning for reevaluation. In a study conducted in Hong Kong, among individuals who had close contact with an index case for more than 8 hours for at least 1 month, the TST positivity rate was 60.6%, while for those with limited contact, the rate was 3.1%.^[40]^ Similarly, in our study, all contact patients had close contact with the index case for more than 8 hours.

Literature shows that TB reactivation can sometimes occur even with isoniazid prophylaxis. According to a study by Çağatay et al, among 583 patients receivingizoniazidchemoprophylaxis, active TB was detected in 5 patients.^[41]^ In our study, despite receiving izoniazid prophylaxis, active TB developed in 7 patients (1.6%). This may be due to factors such as treatment noncompliance, drug interactions, development of resistance, or occurrence of side effects, leading to low effectiveness in prevention.

The limitations of our study include its retrospective design, the possibility of incomplete data in patient records, and the fact that it was a single-center study, which limits its ability to provide nationwide information. Tuberculosis remains an important disease both globally and in our country, and LTBI screening is essential for preventing progression to active disease and contributing to tuberculosis control. Therefore, it is crucial to take appropriate measures, follow-up, and administer effective treatment to at-risk groups. In our study, prophylaxis was administered to 7 (1.6%) patients. The low chemoprophylaxis rate in our study population emphasizes the importance of diagnosing and monitoring LTBI. Our study was not a retrospective evaluation of the incomplete aspects of latent tuberculosis infection diagnosis and treatment, but we believe that broader studies will yield clearer results.

## Author contributions

**Data curation:** Olcay Aycicek, Ezgi Ongoz.

**Formal analysis:** Olcay Aycicek, Ezgi Ongoz, Yilmaz Bülbül.

**Methodology:** Olcay Aycicek.

**Project administration:** Olcay Aycicek.

**Writing – original draft:** Olcay Aycicek, Ezgi Ongoz, Yilmaz Bülbül.

**Writing – review & editing:** Olcay Aycicek, Funda Öztuna, Tevfik Özlü.
